# The genetic variants in 3’ untranslated region of voltage-gated sodium channel alpha 1 subunit gene affect the mRNA-microRNA interactions and associate with epilepsy

**DOI:** 10.1186/s12863-016-0417-y

**Published:** 2016-07-29

**Authors:** Tian Li, Yaoyun Kuang, Bin Li

**Affiliations:** 1 Key Laboratory of Neurogenetics and Channelopathies of Guangdong Province, The Ministry of Education, Institute of Neuroscience, The Second Affiliated Hospital of Guangzhou Medical University, Guangzhou, 510260 Guangdong Province China; 2 Center for Cognitive Neurology, New York University Langone Medical Center, New York, NY 10016 USA; 3 Silver School of Social Work, New York University, New York, NY 10003 USA

**Keywords:** Epilepsy, Untranslated region, microRNA, SNP, Haplotype

## Abstract

**Background:**

mRNA expression in a cell or subcellular organelle is precisely regulated for the purpose of gene function regulation. The 3’ untranslated region (3’UTR) of mRNA is the binding target of microRNA and RNA binding proteins. Their interactions regulate mRNA level in specific subcellular regions and determine the intensity of gene repression. The mutations in the coding region of voltage-gated sodium channel alpha 1 subunit gene, *SCN1A*, were identified in epileptic patients and confirmed as causative factors of epilepsy. We investigated if there were genetic variants in 3’UTR of *SCN1A*, affecting the microRNA-mRNA 3’UTR interaction and *SCN1A* gene repression, potentially associated with epilepsy.

**Results:**

In this case–control study, we identified twelve variants, NM_001202435.1:n.6277A > G, n.6568_6571del, n.6761C > T, n.6874A > T, n.6907 T > C, n.6978A > G, n.7065_7066insG, n.7282 T > C, n.7338_7344del, n.7385 T > A, n.7996C > T, and n.8212C > T in 3’UTR of *SCN1A* gene. We found that the variant of n.6978A > G in all our samples was completely mutated (G/G). In male group, T allele in n.7282 T > C was associated with epilepsy, while C allele was significantly less frequent in epileptic patients than in normal males (OR 0.424). Consequently, the haplotype “CTTACATGACGA” / “CT***C***TA” was significantly less frequent in male epileptic patients (0.173) than in normal males (0.305). The frequency of haplotype block found in females, "TTTAACA", "TTCAACA", and "CTTAACA" was 0.499, 0.254 and 0.234 respectively. Within STarMir model analysis, the “CTCTA” haplotype showed significantly higher site accessibility to microRNA targeting and higher downstream sequence accessibility for nonconserved binding than that of other haplotypes. Overall, the male genotypes have the higher accessibility of the downstream 30nt block of nonconserved site than the female genotypes.

**Conclusions:**

NM_001202435.1:n.7282 T > C is the genetic variant associated with epilepsy in males, and the related haplotype “CTTACATGACGA” / “CT*C*TA” in the region of chr2: 165991297–165989081, which has high site accessibility for microRNA binding, is the genetic protective factor against epilepsy in males. In female subset, the frequencies of haplotype block "TTTAACA", "TTCAACA", and "CTTAACA" were 0.499,0.254 and 0.234 respectively. Alleles and haplotypes distribution did not differ in female cases in comparison to female controls.

**Electronic supplementary material:**

The online version of this article (doi:10.1186/s12863-016-0417-y) contains supplementary material, which is available to authorized users.

## Background

mRNA stability, transport, and local translation are critical for gene function regulation. The mRNA intrinsic sequence of a particular gene and other intracellular factors determines the half-life of the mRNA. MicroRNA (miRNA) binds to a site or a part of sequences in three prime untranslated region (3’UTR) of mRNA, destabilizes mRNA, and represses the targeted gene translation [1]. The variant sequences in 3’ UTR alter the binding feature of miRNA, and influence gene repression process. For example, some SNPs in the responsible genes (*IL23R*, *LCE3D*, et al.) destroyed or created miRNA binding sites and were associated with the clinical psoriasis phenotypes [2]. In a group of schizophrenia patients, rs3219151 (C > T, *GABRA6*) was identified and related to the decreased risk for schizophrenia [3]. miRNA works in a particular way for activity-dependent regulation of mRNA stability and translation [4, 5]. The variants in miRNA coding genes altered miRNA expression, processing, function, and then associated with diseases. Studies showed that rs353291 in miR-145 associated with breast cancer [6], rs11614913 in miR-196a2 associated with bladder cancer [7]. The local concentrations of miRNA and RNA binding proteins determine the binding site occupancies, which in turn regulate mRNA stability and localization, protein production [8].

To analyze the mRNA-miRNA interactions, STarMir is a helpful resource for miRNA studies [9]. It describes many detailed features of predicted sites based on the logistic prediction models [10]. Those features could represent the mRNA-microRNA binding with the probability related to mRNA structure and microRNA binding location. The predicted sites could be distinguished and analyzed with the binding energy, the availability of binding and adjacent sequences, AU percentage in binding sites, and relative starting location of predicted sites. For example, would particular mRNA sequence have higher site availability than others and would the high concentrations of mRNA and microRNA facilitate the mRNA-microRNA binding or induce the competition? STarMir model analysis hypothesizes that miRNA-binding has two steps, nucleation, and hybrid elongation, and both of them requires energy [11]. The power of distinct binding sites displays the difference of each mRNA-miRNA interaction, due to the variant sequence in mRNA binding sites. It is novel to analyze the whole 3’UTR sequences and its binding ability to microRNA, with the usage of STarMir parameters. The imitation of microRNA binding to 3’UTR could provide the information that could be investigated for the potential effects of 3’UTR genetic variants on gene repression.

The abnormality in the gene coding voltage-gated sodium channel alpha1 subunit (*SCN1A*) is a causative factor in febrile seizure related epilepsy syndromes, such as Dravet syndrome, and Genetic Epilepsy with Febrile seizure plus [12]. The mutations of the protein-coding region (or exon) of *SCN1A* gene are also pathogenic factors to neuronal hyperexcitability [13]. Another factor that regulates the *SCN1A* expression is also the potentially epileptogenic factor, such as 5’ untranslated region of *SCN1A* gene [14, 15]. We hypothesize that the variant sequence in 3’UTR is also a critical regulatory mechanism through miRNA-binding 3’UTR interactions [16].

## Methods

### Study subjects

We enrolled 101 epileptic patients and 126 healthy individuals in 2004 through 2010 in The Second Affiliated Hospital of Guangzhou Medical University. The clinical diagnosis of epilepsy or epilepsy syndrome was based on the criteria of the Commission on Classification and Terminology of the International League Against Epilepsy (ILAE) (1981, 1989). All individuals enrolled in this study were not related with one another by the family relationship or consanguinity.

### Genotyping and allele analysis of variants

The genomic DNA was extracted from peripheral white blood cells of participants with guanidine/SDS method (see Additional file 1: Supplemental material 1). We applied the final genomic DNA collection directly in PCR experiment for target fragments replication (3’UTR in *SCN1A* gene). The four pairs of primers for genomic DNA replication were listed below, wp615-5’-TGATCTGACCATGTCCACTGC, wp616-5’-CCCTCATGCAAACCACGAC (680 bp); wp617-5’-TTTTGTAAACGAAGTTTCTGTTGAG, wp618-5’-GAAACCAGATACAGCAGCATGG (732 bp); wp619-5’-TGTAGAGTGCAAGCTTTACACAGG, wp620-5’-GAATCGTGAACCTATTTTGCTCC (601 bp); wp621-5’-CACAATCACTTTTCTTACTTTCTGTCC, wp622-5’-CCTTCTCCCCCAATTTGTAATG (660 bp). We then sent the PCR products to BGI Guangzhou Office (ABI 3730xl sequencer) for sequencing. If the sequencing files indicated the deletion or insertion mutations, we would use molecular cloning method to amplify the single genomic DNA chain within cloning vectors for re-evaluation. The variants identified by genotyping were summarized in male and female group respectively. The number of “aa”, “ab”, and “bb” genotypes were summed into chi-square test table for genotype-associated study. At the same time we calculated the frequency and number of two alleles (a or b) with the formula (a = 2*aa + ab; b = 2*bb + ab) and summarized them in another chi-square test table for allele-associated study. The *p*-value less than 0.05 was the criteria for the statistically significant difference.

### Haplotype analysis

The findings of genetic variants from male and female individuals were summarized into two tables (Additional file 2: Table S1 and Table S2) with Haploview program [17] Linkage Format (*.ped). The form or file listed the variables of family ID (enrollment ID into the study), Individual ID (case ID in Epilepsy Center or in Healthy Center), Father ID (zero, independent without family relationship between subjects), Mother ID (zero), gender (1 means male, 2 means female), affection status (1 means unaffected, 2 means affected or with epilepsy), and the alleles labeled with two numbers from zero to four (1 = A, 2 = C, 3 = G, 4 = T, 0 = missing data or deletion). By choosing “Four Gamete Rule” to define blocks, the haplotype data were displayed with frequency and chi-square value, the association *p*-value.

### STarMir input, output, and data analysis

miRNA data from Landgraf et al. [18] were downloaded and the approximate 50 miRNAs mostly expressed in each part of CNS (hippocampus, frontal cortex, midbrain, and cerebellum) were input into the miRbase database (http://www.mirbase.org//cgi-bin/starmirtest2.pl/) for mature 22 nt-miRNA form. In the STarMir web (http://sfold.wadsworth.org/cgi-bin/starmirtest2.pl), we uploaded one 50-miRNA set miRNA files into “microRNA sequence(s), manual sequence entry”. The 3’UTR sequence files were modified with background genetic variant, n.6978A > G (G/G), and each of seven haplotypes, two deletions, one insertion, or one wild-type (reference sequence). The modified sequence files were input into the option of “single target sequence, manual sequence entry”. By choosing “V-CLIP based model (human)”, “Human (homo sapiens)”, and “3’UTR”[10], the following parameters [19] would be displayed in the output window and ready for further analysis: “LogitProb”, the probability of the site being a miRNA-binding site as predicted by STarMir logistic model. “site_position”, start to end position of the predicted miRNA target region in mRNA; “seed_position”, start to end position of the target sub-region in mRNA complementary to the miRNA seed (corresponding to positions 2-7/8 of the miRNA); “seed_type”, 6mer, offsite 6mer, 7mer-A1, 7mer-m8, and 8mer seed sites described in Bartel 2009 [20]; “site_access”, the structural accessibility as calculated by the average probability of a nucleotide in mRNA being single-stranded for binding the nucleotides in microRNA; “seed_access”, the structural accessibility as calculated by the average probabilities being single-stranded of the nucleotides in the target sub-region of mRNA complementary to the miRNA seed; “upstream_access (#nt)”, the structural accessibility described by the average probabilities of single-stranded nucleotides in upstream block of the predicted binding site (# is the block size); “dwstream_access (#nt)”, the structural accessibility displayed by the average of single-stranded probabilities for the downstream block of the predicted site; “upstream_AU (#nt)”, the percentage of AU for the upstream block of the binding site (# is the block size); “dwstream_AU (#nt)”, the percentage of AU for the downstream block of the binding site (# is the block size); “site_location”, the location of the predicted site relative to the start and end along the entire length of sequence (for 3’UTR, zero represents the 5’ end and one accounts for the 3’ end); ΔGhybrid, the stability of miRNA:target hybrid as calculated by RNAhybrid [21] in Rehmsmeier et al. 2004; ΔGnucl, the potential nucleation for miRNA:target hybridization [10]; ΔGtotal, the total energy change during the entire process of hybridization [11]. We filtered the output data with criteria of LogitProb of 0.5 or higher [10] and ΔGhybrid of −15 kcal/mol or lower [22]. We compared the means of those parameters in each genotype group with one-way ANOVA or Kruskal-Wallis test. We also added the miRNA copy number [18] and mRNA expression amount (reads per kilobase transcript per million reads, RPKM, http://www.gtexportal.org/home) [23] into the data pool for the correlation and regression analysis with STarMir parameters.

### Statistical analysis

Manual chi-square table was applied to describe the distribution of common genetic variants in case/control study, to calculate and compare the genotype frequency and allele frequency in case group and control group. Fisher’s exact test was used to analyze the distribution of rare genetic variants in case and control groups. Haploview software 4.2 was applied to calculate the haplotype frequency in groups and the chi-square value, *p*-value in haplotype association test. Using IBM SPSS Statistics 23 (IBM Corporation, Armonk, NY, USA), we compared the means, standard deviation (SD), standard errors of means (SEM) of STarMir parameters in “genotype” groups (11 genotypes) with one-way ANOVA, and “Dunnett T3” in Post Hoc. If the data of some STarMir parameters did not pass the “homogeneity for variants” test (*p* < 0.05) the non-parameter test, “independent samples Kruskal-Wallis test” was used to compare the mean ranks of multiple genotype groups. “Bivariate Correlation” and “Spearman” were applied in the correlation analysis (*p* < 0.05 and r ≠ 0) with miRNA expression weight or mRNA expression quantities as “x” variable, and other STarMir parameters as “y” variable. In linear regression analysis, “miRNA expression weight” or “mRNA expression” was “Independent(s)” and other significantly-correlated STarMir parameters were “Dependent”, *p* < 0.05 in “ANOVA” table to define the significant regression. We used the B values of constant and mRNA_expr to fill the linear vector form (y = ax + b).

## Results

### Study subjects

We collected *SCN1A* 3’UTR genotyping data from 101 epileptic patients (52 males and 43 females) and 126 controls (59 males and 68 females). All patients, when enrolled in this study, were older than two years old. In the male subgroup, 15 (29 %) patients had a positive history of febrile seizures. 28 (53.8 %) patients were at the age of 2–9 years, 11 (21.1 %) patients were at the age of 10–19 years, and 7 (13.5 %) patients were at the age of 20–29 years. In the female subgroup, eight (18.6 %) patients had a positive history of febrile seizures. 18 (41.9 %) patients were at the age of 2–9 years. 13 (30.2 %) patients were at the age of 10–19 years. And five (11.7 %) patients were at the age of 20–29 years. In the male healthy control group, 29 (49.2 %) males were at the age of 20–29 years. Among female controls, three (4.9 %) females were at the age of 10–19 years and 45 (73.8 %) females were at the age of 20–29 years. All participants were Chinese Han, and most of them lived in southern China.

### *SCN1A*-3’UTR Genotyping and association analysis of single variants

The genomic DNA variants, NM_001202435.1:n.6277A > G, n.6568_6571del, n.6761C > T, n.6874A > T, n.6907 T > C, n.6978A > G, n.7065_7066insG, n.7282 T > C, n.7338_7344del, n.7385 T > A, n.7996C > T, and n.8212C > T were revealed in the 2.2kbp-length region (chr2:165991297–165989081) of 3’ UTR of *SCN1A* gene (*SCN1A*_v001). The distribution of n.7282 T > C was significantly different in the male group: its genotype, CC, and CT, was much less frequent in male patients than in male controls (OR 0.424, 95 % CI [−1.61, −0.11], *p* < 0.05). Other two common variants, n.7996C > T, CC and CT (OR 0.875, 95 % CI [−0.89, 0.62]), and n.8212C > T, CT and TT (OR 0.77, 95 % CI [−1.12, 0.60]), did not significantly distribute differently between cases and controls. In female subset, three variants were distributed relatively even in the patient and control group, n.7282 T > C (OR 1.50, 95 % CI [−0.36, 1.17]), n.7996C > T (OR 0.91, 95 % CI [−0.86, 0.68]), n.8212C > T (OR 1.03, 95 % CI [−0.94, 1.01]). The genetic variant n.6978A > G was fully deviated (G/G, 100 %) from that of the reference (A/A). We set its genotype (G/G) as the sample background genotype in the miRNA-3’UTR interaction study. The variants frequencies of n.6277A > G, n.6568_6571del, n.6761C > T, n.6874A > T, n.6907 T > C, n.7065_7066insG, n.7338_7344del, n.7385 T > A, were quite low, one or two cases in some gender group (male group or female group). The *p*-values from their Fisher’s exact tests showed that none of them was associated with case/control differences (Additional file 2: Table S4). Besides the genotype association test, we also calculated the single allele frequency and allele association test between case and control group. Consistently, the C allele of n.7282 T > C in male patients (0.191) was significantly less frequent (*p* < 0.05) than that in male controls (0.305). We displayed the detailed chi-square test in Table 1.

**Table 1:** The distribution of n.7282 T > C, n.7996C > T, n.8212C > T in males and females case/control groups

**n.7282 T > C**		**CC+CT**	**TT**	**C**	**T**	**C frequency**
	Female patients	24	22	25	67	0.272
	Female controls	28	40	34	102	0.250
			*p > 0.05*		*p > 0.05*	
	Male patients	21	34	21	89	0.191
	Male controls	35	24	36	82	0.305
			***p*** **<0.05**		***p*** **<0.05**	
**n.7996C > T**		**CC+CT**	**TT**	**C**	**T**	
	Female patients	18	28	18	74	0.196
	Female controls	26	42	29	107	0.213
			*p > 0.05*		*p > 0.05*	
	Male patients	21	34	22	88	0.200
	Male controls	24	34	25	91	0.216
			*p > 0.05*		*p > 0.05*	
**n.8212C > T**		**CC**	**CT+TT**	**C**	**T**	**T frequency**
	Female patients	38	8	84	8	0.087
	Female controls	55	13	122	14	0.103
			*p > 0.05*		*p > 0.05*	
	Male patients	40	15	94	16	0.145
	Male controls	45	13	102	14	0.121
			*p* > 0.05		*p* > 0.05	

### Haplotype analysis in case/control study

Seven major haplotypes in male (4 haplotypes) and female (3 haplotypes) subset were identified (Table 2). There were “CTTTA” (0.445), “CTCTA” (0.241), “CCTTA” (0.186), and “TTTTA” (0.114) in male subset. The most frequent two haplotypes had the higher distinction between cases and controls. The frequency of “CTTTA” was 0.490 in male patients and 0.361 in male controls. But the haplotype association test showed that only the frequency of haplotype “CTCTA” was significantly lower in male patients (0.173) than in male controls (0.305) (*p* = 0.026). In the female subset, the frequency of haplotype block “TTTAACA”, “TTCAACA”, and “CTTAACA” was 0.499, 0.254, and 0.234 respectively. They distributed quite even between patients and controls. In female patients, the frequency of three haplotypes were 0.512, 0.279, and 0.209, while in female controls, the frequency of three haplotypes were 0.490, 0.239, and 0.249 respectively. In the female subset, there was no statistical difference in case/control distributive frequency. (Table 2).

**Table 2:** The Haploview association analysis of blocks in the male and female group. *marked the data point with statistically significant difference in chi-square test (*p*<0.05)

	Full block	Abbre.	Freq	Case, control counts	Case, control ratio	Chi square	*p* value
Male							
	CTTATATGACGA	CTTTA	0.445	51:53,47:69	0.490,0.361	1.612	0.2042
	**CTTACATGACGA**	**CTCTA**	**0.241**	**18:86,35:81**	**0.173,0.305**	**4.963**	0.0259*
	CCTATATGACGA	CCTTA	0.186	22:82,19:97	0.212,0.206	0.824	0.3639
	TTTATATGACGA	TTTTA	0.114	13:91,12:104	0.125,0.103	0.253	0.6151
Female							
	TTATATGACGA	TTTAACA	0.499	44:42,67.7:70.3	0.512,0.490	0.095	0.7578
	TTACATGACGA	TTCAACA	0.254	24:62,33:105	0.279,0.239	0.445	0.5045
	CTATATGACGA	CTTAACA	0.234	18:68,34.3:103.7	0.209,0.249	0.459	0.498

### STarMir model analysis of *SCN1A*-3’UTR haplotype and miRNA interaction

**Fig. 1:**
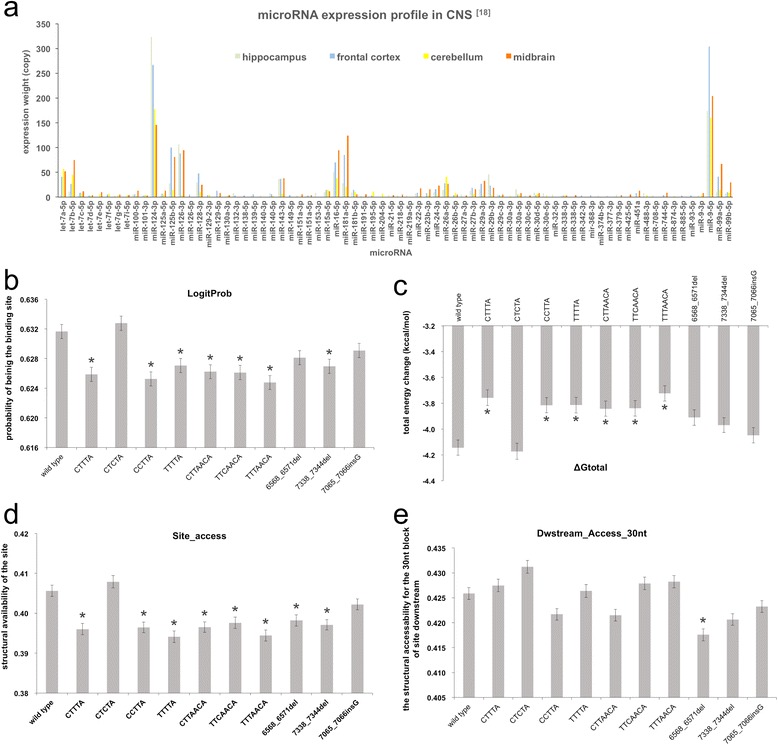
miRNA expression profile in CNS and the means of STarMir parameters in genotype groups. **a** According to Landgraf P et al., 2007 [18], the four parts of CNS, hippocampus, frontal cortex, cerebellum, and midbrain, have distinctive miRNA expression profile. The approximate 50 miRNAs most expressed in each part were described in a color-coded histogram with copyright permission (Additional file 1: Table S4). **b** the means of LogitProb in genotype groups were significantly different. * represents that the mean of LogitProb in the genotype group was significantly different from that of wild type group. **c** the means of ΔGtotal in genotype groups were significantly different. * represents that the mean of ΔGtotal in the genotype group was significantly different from that of wild type group. **d** the means of site_access in genotype groups were significantly different. * represents that the mean of site_access in the genotype group was significantly different from that of wild type group. **e** the means of Dwstream_access_30 nt in genotype groups were significantly different. * represents that the mean of Dwstream_access_30 nt in the genotype group was significantly different from that of wild type group

We summarized the 50 highly-expressed mature miRNAs in four parts of the central nervous system (CNS) in Fig. 1a, Additional file 2: Table S5.

1. STarMir parameter comparison among genotypes

STarMir parameters described the mRNA-miRNA binding features, which could be largely determined by the intrinsic sequence of mRNA (3’UTR) [1]. In order to reveal the different effect of significant genotype or haplotype on mRNA-miRNA binding, we compared the means of STarMir parameters from each genotype group. And we found that the significant difference (*p* < 0.05) in the binding probability (LogitProb), site accessibility (site_access), the total energy change (ΔGtotal), and the single-strand probabilities of 30 nt block of nonconserved binding site downstream (dwstream_access_30nt) due to the genotype variants (Table 3). The genotype “CTCTA” had the highest site_access (0.408 ± 0.128), LogitProb (0.633 ± 0.081), ΔGtotal (−4.172 ± 5.175 kcal/mol), and dwstream_access_30 nt (0.431 ± 0.001), even higher than wild-type genotype (0.406 ± 0.117, 0.632 ± 0.079, −4.143 ± 4.935 kcal/mol, 0.426 ± 0.001). The genotype “TTTAACA” has the lowest LogitProb (0.625 ± 0.001) and ΔGtotal (−3.723 ± 0.060). The genotype “TTTTA” had the lowest site_access (0.394 ± 0.001) and the genotype “6568_6571del” had the lowest dwstream_access_30 nt_nonconserved (0.421 ± 0.001) (Fig. 1b–e). The means of STarMir parameters of a single part of CNS (hippocampus, frontal cortex, cerebellum, or midbrain) were in Additional file 2: Table S6.

**Table 3:** The mean ± SD (standard deviation) of STarmir parameters in 11 genotype groups

	LogitProb	ΔGhybrid	ΔGnucl	ΔGtotal	site_access	Upstream_Access_10nt_nonconserved	Dwstream_Access_30nt_nonconserved	Upstream_AU_30nt	Dwstream_AU_30nt	Site_Location	Seed_Access	Upstream_Access_15nt_conserved	Dwstream_Access_10nt_conserved
Wild type	0.632 ± 0.079	−17.378 ± 2.116	−1.866 ± 1.802	−4.143 ± 4.935	0.406 ± 0.117	0.407 ± 0.163	0.426 ± 0.099	0.663 ± 0.116	0.670 ± 0.101	0.457 ± 0.289	0.385 ± 0.169	0.419 ± 0.113	0.382 ± 0.122
CTTTA	0.626 ± 0.079	−17.397 ± 2.129	−1.789 ± 1.774	−3.757 ± 5.011	0.396 ± 0.117	0.411 ± 0.166	0.427 ± 0.103	0.661 ± 0.116	0.669 ± 0.102	0.450 ± 0.288	0.350 ± 0.165	0.462 ± 0.162	0.393 ± 0.142
CTCTA	0.633 ± 0.081	−17.371 ± 2.107	−1.870 ± 1.894	−4.172 ± 5.175	0.408 ± 0.128	0.412 ± 0.175	0.431 ± 0.107	0.660 ± 0.116	0.669 ± 0.102	0.448 ± 0.287	0.369 ± 0.191	0.445 ± 0.148	0.383 ± 0.137
CCTTA	0.625 ± 0.079	−17.409 ± 2.135	−1.829 ± 1.719	−3.815 ± 4.867	0.396 ± 0.110	0.405 ± 0.155	0.422 ± 0.096	0.662 ± 0.116	0.669 ± 0.102	0.458 ± 0.289	0.359 ± 0.165	0.448 ± 0.130	0.387 ± 0.135
TTTTA	0.627 ± 0.079	−17.400 ± 2.124	−1.824 ± 1.790	−3.814 ± 5.027	0.394 ± 0.119	0.408 ± 0.166	0.426 ± 0.107	0.662 ± 0.116	0.669 ± 0.102	0.453 ± 0.289	0.335 ± 0.169	0.458 ± 0.149	0.392 ± 0.131
CTTAACA	0.626 ± 0.078	−17.407 ± 2.135	−1.823 ± 1.711	−3.841 ± 4.851	0.397 ± 0.110	0.406 ± 0.155	0.422 ± 0.096	0.663 ± 0.116	0.669 ± 0.102	0.459 ± 0.290	0.362 ± 0.137	0.446 ± 0.125	0.393 ± 0.122
TTCAACA	0.626 ± 0.080	−17.394 ± 2.126	−1.824 ± 1.782	−3.839 ± 5.007	0.398 ± 0.120	0.410 ± 0.167	0.428 ± 0.104	0.659 ± 0.117	0.668 ± 0.102	0.450 ± 0.287	0.346 ± 0.156	0.459 ± 0.142	0.392 ± 0.131
TTTAACA	0.625 ± 0.078	−17.397 ± 2.129	−1.782 ± 1.752	−3.724 ± 4.940	0.394 ± 0.115	0.410 ± 0.166	0.428 ± 0.103	0.661 ± 0.116	0.668 ± 0.102	0.450 ± 0.287	0.357 ± 0.167	0.461 ± 0.157	0.396 ± 0.138
6568_6571del	0.628 ± 0.078	−17.401 ± 2.130	−1.841 ± 1.788	−3.910 ± 4.956	0.398 ± 0.116	0.408 ± 0.162	0.418 ± 0.099	0.663 ± 0.116	0.668 ± 0.103	0.460 ± 0.290	0.347 ± 0.147	0.433 ± 0.132	0.376 ± 0.135
7338_7344del	0.627 ± 0.079	−17.408 ± 2.131	−1.844 ± 1.681	−3.969 ± 4.686	0.397 ± 0.106	0.406 ± 0.149	0.421 ± 0.095	0.663 ± 0.116	0.669 ± 0.103	0.459 ± 0.290	0.369 ± 0.127	0.457 ± 0.119	0.393 ± 0.109
7065_7066insG	0.629 ± 0.079	−17.394 ± 2.124	−1.843 ± 1.741	−4.048 ± 4.858	0.402 ± 0.112	0.406 ± 0.158	0.423 ± 0.097	0.662 ± 0.116	0.669 ± 0.102	0.458 ± 0.290	0.373 ± 0.164	0.431 ± 0.125	0.382 ± 0.127

2. The description and comparison of conserved sites and non-conserved sites

**Fig. 2:**
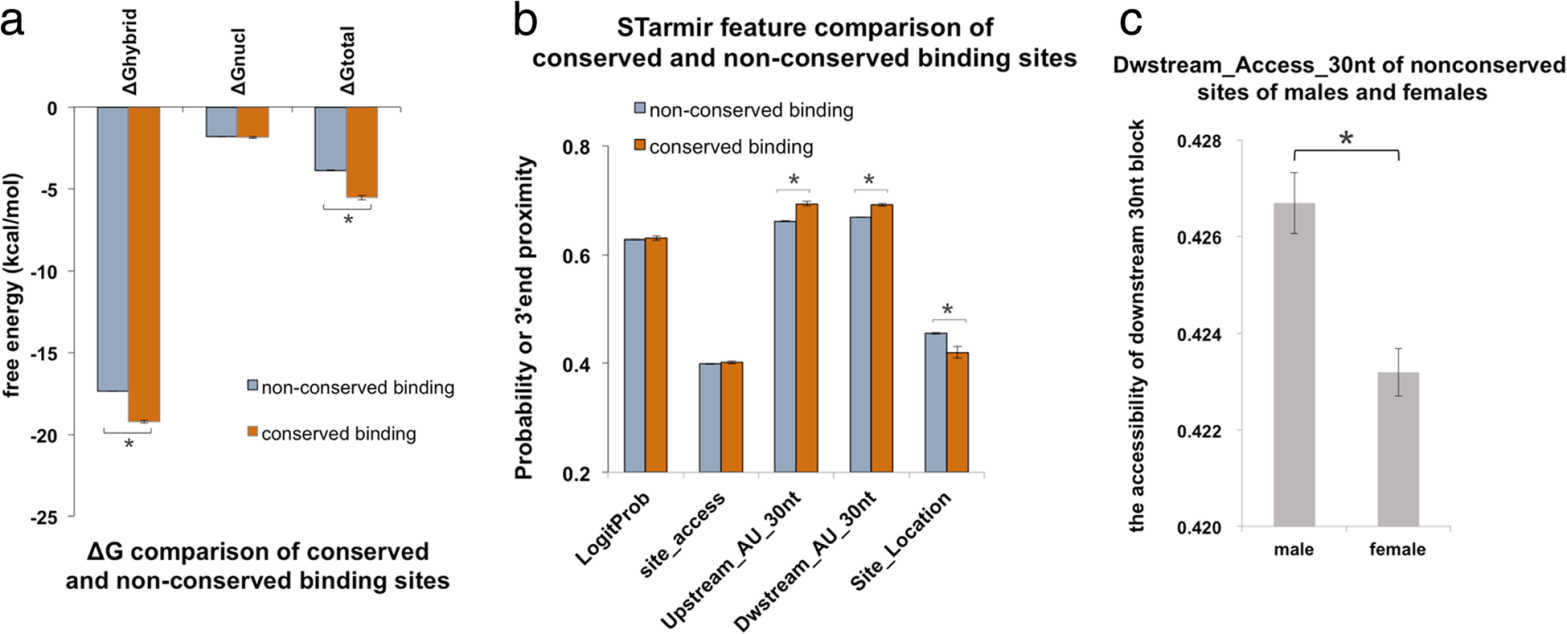
STarMir parameter comparison between conserved/ nonconserved sites and gender groups. **a** the free energy change in conserved and nonconserved binding. * represents that the mean of the free energy parameter in nonconserved sites was significantly different from that of conserved sites. **b** the comparison of STarMir parameters commonly used in both conserved and nonconserved sites. * represents that the mean of the marked STarMir parameter in nonconserved sites was significantly different from that in conserved sites. **c** the accessibility of downstream 30 nt block of the nonconserved site in male and female groups. * represents that the mean of the Dwstream_access_30nt_nonconserved of male genotypes was significantly different from that of female genotypes

The numbers of predicted conserved sites for wild-type 3’UTR were 30, 25, 20 and 21 from the data pools of the hippocampus, frontal cortex, cerebellum, and midbrain (Additional file 2: Table S7). The miRNA interaction with 3’UTR variants changed with decreased numbers of conserved sites (Additional file 3). Binding with variant genotype, some miRNA was losing its conserved site, while alternative miRNA gained a new site for compensation. The frequently lost sites included miR-27b-3p at nt398-414, miR-9-5p at nt962-987, miR-130a-3p at nt1836-1854, and miR-29a-3p at nt766-786. The site of miR-30a-5p at nt408-429 and miR-204-5p at nt275-299 were the common sites for compensation (Additional file 2: Table S7). The parameters of upstream_access_15nt and dwstream_access_10nt of conserved sites were not significantly different between wild-type and other genotypes (Table 3, Additional file 2: S6). The number of predicted nonconserved binding sites of wild-type (WT) haplotype was 1610, 1735, 1727, and 1730 from four parts of CNS. The conserved binding sites were much less than the nonconserved binding sites. And hence the two conserved site parameters, upstream_access_15nt and dwstream_access_10nt, had less powerful statistical results in genotype comparison. With the comparison of conserved and nonconserved binding site, We found that the conserved sites had higher ΔGhybrid (−19.222 ± 0.088 kcal/mol), higher ΔGtotal (−5.546 ± 0.142 kcal/mol), higher upstream_AU_30 nt (0.693 ± 0.004), higher dwstream_AU_30 nt (0.692 ± 0.003), lower site_location (0.420 ± 0.010), compared to the nonconserved binding (ΔGhybrid −17.371 ± 0.001 kcal/mol, ΔGtotal −3.890 ± 0.018 kcal/mol, upstream_AU_30 nt 0.661 ± 0.0004, dwstream_AU_30 nt 0.669 ± 0.0004, site_location 0.455 ± 0.001) (Fig. 2e,f).

3. The comparable means of StarMir parameters from gender-deviated genotypes

We calculated the haplotypes from male and female groups separately and correspondingly the male group and the female group had distinctive haplotypes, four haplotypes from males, three haplotypes and three insertion/deletions from females. After the STarMir parameters were calculated based on the male genotypes (CTTTA, CTCTA, CCTTA, and TTTTA) and female genotypes (CTTAACA, TTCAACA, TTTAACA, 6568_6571del, 7065_7066insG, and 7338_7344del). We found that dwstream_access_30 nt_nonconserved was significantly different (*p* < 0.001) between males (0.427 ± 0.103, mean ± SD) and females (0.423 ± 0.099) (Fig. 2 g, Additional file 2: Table S9).

4. miRNA and mRNA expression level affects miRNA-mRNA (3’UTR) interaction

**Fig. 3:**
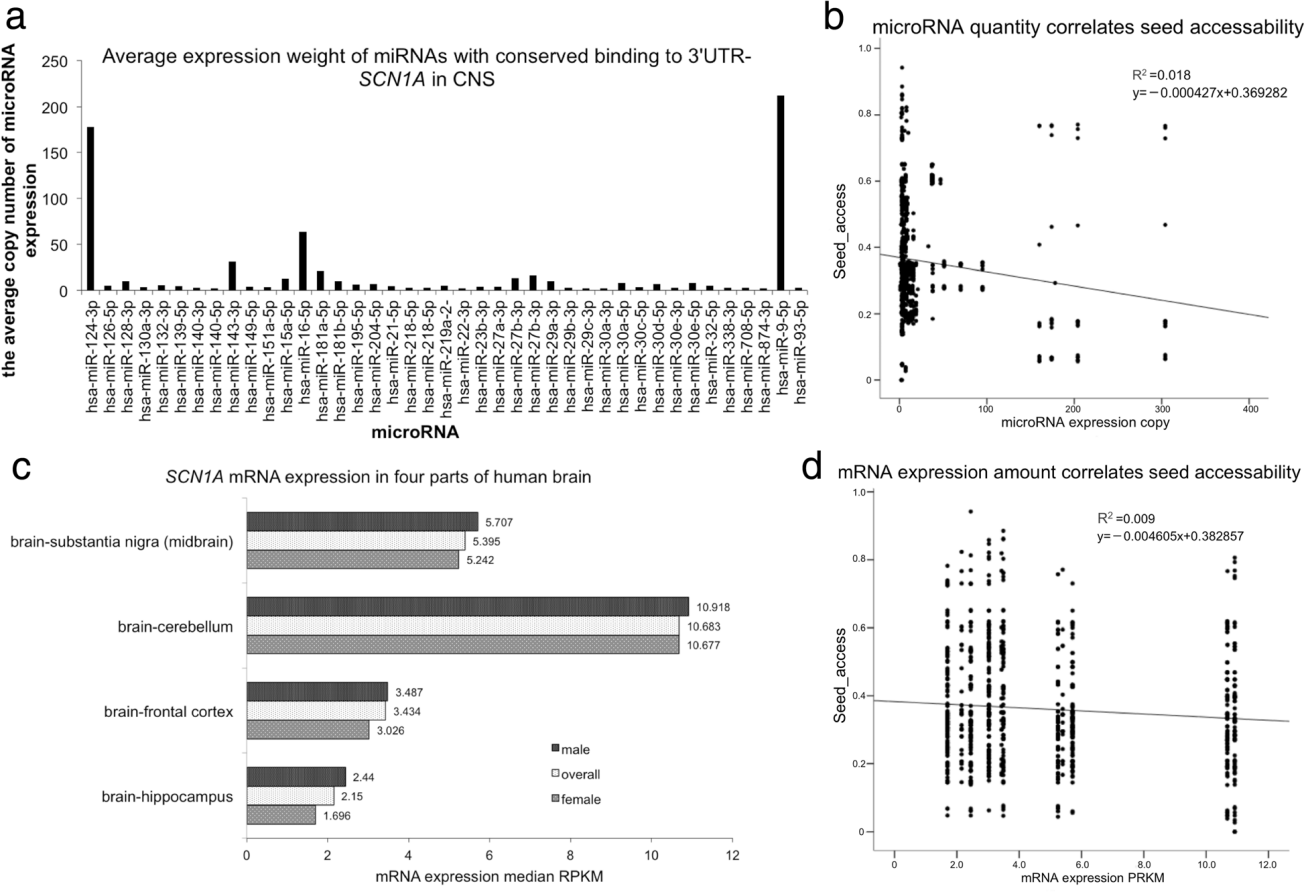
The correlation and regression analysis of miRNA expression or mRNA baseline expression with the seed accessibility of miRNA conserved binding sites. **a** the expression copies of microRNAs binding the predicted conserved sites of 3’UTR in *SCN1A* gene in all four parts of CNS. **b** the correlation and linear regression illustration of seed accessibility with miRNA expression copies. The linear regression constant and vector form were shown on the upper right part of the figure. **c** the *SCN1A* mRNA expression profile in the human brain, according to the GTEx Consortium 2015 [23]. The mRNA expression RPKM of male group, female group, and overall average were revealed in four parts of the human brain. **d** the negative correlation and regression relationship of seed accessibility with baseline mRNA expression level. The regression constant and vector form were shown in the upper right part of the figure

Besides the intrinsic mRNA-3’UTR sequence, we investigated if miRNA expression quantities affected the miRNA-mRNA (3’UTR) *SCN1A* interaction significantly. Using correlation and sequential regression analysis, we found that miRNA expression copies correlated LogitProb, ΔGhybrid, ΔGnucl, upstream_AU_30 nt, dwstream_AU_30 nt, and seed_access with linear regression (both tests reached *p* < 0.05) significantly. The seed_access had the highest correlation coefficient (*r* = −0.117) among those parameters. But the correlation was in low level ($\left|r\right|<0.4)$ (Table 4). On the other hand, we investigated how the baseline mRNA expression affected the miRNA-mRNA (3’UTR) interactions. The *SCN1A* mRNA expression profile in human brain was 2.15 RPKM in hippocampus, 3.434 RPKM in frontal cortex, 10.683 RPKM in cerebellum, and 5.395 RPKM in midbrain [23] (Fig. 3c). Using Spearman’s correlation and linear regression analysis, we found that mRNA expression baseline significantly correlated dwstream_access_30 nt_nonconserved, site_location, seed_access, and dwstream_access_10nt_conserved with linear regression relationship (both tests resulted in *p* < 0.05). The dwstream_access_10nt_conserved had the highest correlation coefficient, *r* = 0.072, among the four parameters. Therefore, the seed_access had the negative correlation with both mRNA expression baseline (*r* = −0.068), and microRNA expression copy number (*r* = −0.117) (Table 4, Fig. 3).

**Table 4:** the correlation and linear regression analysis of mRNA and microRNA

	y=	Vector form	Correlation coefficient	Regression R square linear	Regression p
x = microRNA expression copy	LogitProb	y = −0.000047x + 0.628775	−0.024	0.000888	*p < 0.001*
	ΔGnucl	y = −0.00029x-1.823338	0.023	0.000832	*p < 0.001*
	ΔGhybrid	y = 0.001226x-17.425679	−0.046	0.000067	*p = 0.025*
	upstream_AU_30nt	y = −0.000032x + 0.662505	−0.021	0.000191	*p < 0.001*
	dwstream_AU_30nt	y = 0.000025x + 0.668299	0.008	0.000146	*p = 0.001*
	seed_access	y = −0.000427x + 0.369282	−0.117	0.01815	*p < 0n001*
x = mRNA expression level	dwstream_access_30nt_nonconserved	y = −0.000327x + 0.426487	−0.008	0.00012	*p* = 0.003
	site_location	y = 0.000632x + 0.451115	0.008	0.000054	*p* = 0.044
	seed_access	y = −0.004605x + 0.382857	−0.068	0.008944	*p* = 0.002
	dwstream_access_10nt_conserved	y = 0.003130x + 0.372415	0.072	0.006371	*p* = 0.01

## Discussion

Gene mutations in a coding region caused amino acid replacement, deletion, abnormal protein structure, or truncated/incomplete protein sequence. In non-coding regions of DNA sequence, the variants may exist in order to regulate the gene product quantity or preserve characteristic information [4]. In the genotyping results, n.6978A > G was fully deviated from the reference (A/A). It may indicate that our subjects’ *SCN1A* gene was originated from a narrow genetic source with distinctive inherent background (racial factor), or our subjects are under the influence of the small range of residency area or of similar social factors. The allele of n.7282 T > C had significantly lower frequency of C in male patients than that in normal males. Consequently, the frequency of haplotype “CTCTA” was significantly lower in patients group. It indicated that C allele in n.7282 T > C should be a protective factor against epilepsy (OR 0.424). The miRNA-mRNA (3’UTR) interaction STarMir prediction data supported this finding: the structural accessibility of the predicted site in “CTCTA” 3’UTR (0.408 ± 0.128) was as high as that in wild-type (0.406 ± 0.117), while site_access of other genotypes was significantly lower than that of wild-type. Additionally, “CTCTA” genotype had also the higher accessibility of downstream sequence of non-conserved binding sites (0.431 ± 0.107) than wild-type genotype (0.426 ± 0.099). “CTCTA” should be friendly accessible for miRNA binding and gene repression process, which should not be halted or handicapped by the mutated 3’UTR sequences. Instead, the 3’UTR sequences of other genotypes could influence the miRNA-related gene repression negatively. In the female subset, although we identified several novel mutations and recognized the major alternation in STarMir parameters of two deletions and one insertion mutation, due to few mutated case (only one for each mutation) we could not reach the statistically significant difference between female patients and controls. Other mutations were distributed very even in female patients and female controls. On the contrary, in males, there were no severe mutations that changed the microRNA-mRNA (3’UTR) interaction, but the common variants were distributed differently in male patients and male controls, which contributed to the statistically significant and comparative results. In comparison to other studies, the authors analyzed the genetic variants in 3’UTR with other mechanisms on gene regulation, such as AU-rich sequence [24], GAPDH-binding site [25]. Our study could have the limitation of the chi-square test and the fundamental theory, the microRNA-mRNA (3UTR’) interaction, to analyze the genetic variants in 3’UTR.

Overall, the miRNA expression negatively influenced microRNA-mRNA (3’UTR) interaction according to the correlation and regression results on STarMir parameters. The predominantly negative effects of higher miRNA expression were the lower probability of being the site predicted, the lower stability of microRNA:target, the lower percentage of AU in upstream block, and the lower structural accessibility of microRNA-complementary sequence. We might have interpreted that more microRNA would induce the competition among the possible binding sites, the excessive possibility of microRNA:target binding could interfere the existing microRNA:target hybridization, more microRNA would impair the accessibility of microRNA-complementary sequences, and the more microRNA could shift the binding sites to Less AU upstream sites. On the contrary, the mRNA quantities could positively influence the microRNA-mRNA (3’UTR) interaction by promoting 3’end-proximal site binding, and higher downstream sequence of conserved site accessibility. Based on the negative effects of microRNA and mRNA quantity on seed_access, we could reasonably assume that on the physiological baseline, the microRNA expressed in CNS and mRNA of *SCN1A* are relatively excessive to conserved binding of microRNA-mRNA (3’UTR) interactions, which might be an interesting study direction in the future.

STarMir principally uses two free energy values to predict and calculate the microRNA-mRNA binding features [11]. It is believed that STarMir has less putative targets with additional specific threshold condition [26]. It required the input mRNA sequence less than 5000 nt. However, our study subject, *SCN1A* gene, has the long mRNA sequence (NM_001202425.1), 8342 bp. Although STarMir provides the optimal option for mRNA input, the full length of mRNA for complex structural assessment, we were able to input 3’UTR sequence of *SCN1A* gene, a part of mRNA sequence, for binding site prediction. For the purpose of the description of binding sites, STarMir had better performance on our task, providing multiple parameters for the site comparison and analysis. Other microRNA predictive tools, such as PITA [26], and RNAhybrid [21] could not provide those binding features for our purposes.

The diseases of multifactorial inheritance have complex etiology with the function of multiple genes and the complex epigenetic mechanisms are involved. Epilepsy is one of the diseases of multifactorial inheritance. The causative factors include the dysfunctional gene products of *SCN1A*, *GABRA1* (alpha subunit of GABA receptor), and *CHRNA4* (subunits of nicotinic AChr receptor) gene [27]. Many factors are contributing to the gender difference of human brains, such as sex hormone physiology, the fine tune of neuroendocrine system functioning [28, 29], and the environmental and educational interferences. Consistently, the males and females have different mRNA expression baseline of *SCN1A* gene in the brain [23], which further supports our finding and could also be the outcome of gene regulation based on microRNA-mRNA interaction. Generally speaking, our study presented the gender-different data probably resulting from epigenetic mechanism (actively involving environmental factors), and dynamic miRNA-mRNA (3’UTR) interaction. On the other side, it could also be the outcome of human gender-different adaption and selection in the genetic evolution. How the sex factors fine-tune the gene expression and repression or the function of voltage-gated sodium channels would be an attractive topic for clinical scientists and neurobiologists in the future.

## Conclusions

Using case/control association study and STarMir model, we efficiently analyzed the genetic variants in 3’UTR of *SCN1A* gene in epileptic patients and small-sized controls. The male epileptic patients had significantly lower frequency of C allele in n.7282 T > C than normal males, and the OR value was 0.424. The related haplotype “CTCTA” also had the significantly lower frequency in male epileptic patients. The frequencies of haplotype block "TTTAACA", "TTCAACA", and "CTTAACA" in female subset were 0.499,0.254 and 0.234. Their frequencies were not significantly different between case and controls. The STarMir analysis displayed that male haplotype “CTCTA” had high site accessibility and other favorable features for miRNA binding, compared with other genotypes and haplotypes. The 3’UTR or related miRNAs could be the potential targets of therapeutic strategies in the future study of epilepsy.

## Abbreviations

3’UTR, 3’ untranslated region; miRNA, microRNA; *SCN1A*, voltage-gated sodium channel α 1 subunit; SNP, single nucleotide polymorphism; *IL23R,* interleukin 23 receptor; *LCE3D*, late cornified envelope 3D; *GABRA*6, gamma-aminobutyric acid (GABA) A receptor, subunit α 6; *GABRA1,* gamma-aminobutyric acid (GABA) A receptor, subunit alpha 1; *CHRNA4,* subunits of nicotinic acetylcholine receptor.

## Additional files

Additional file 1: Supplemental material. (DOCX 116 kb) Additional file 2: Table S1. Genetic variants and alleles in 3'UTR of *SCN1A*_v001 of male patients and controls (in *.ped file); **Table S2.** genetic variants and alleles in 3'UTR of *SCN1A*_v001 of female patients and controls (in *.ped file); **Table S3.** genetic variants in 3'UTR-*SCN1A* found in study subjects and their locations (in *.info file); **Table S4.** Fisher’s exact test on rare genetic variants for case/control association study; **Table S5.** 50 most expressed miRNA in four parts of CNS; **Table S6.** STarMir parameters of predicted miRNA-binding sites of 3’UTR of *SCN1A* gene in genotype groups; **Table S7.** the frequently lost and compensatory sites for the alteration in conserved sites of miRNAs binding of 3’UTR-*SCN1A* in genotype groups. **Table S8.** the conserved sites of miRNA binding in wild type 3’UTR-*SCN1A.* **Table S9.** the comparison of STarMir parameters between males and females. “S0”-“S14.txt” were the working input files for STarMir analysis. **S0.** wild type (reference) 3’UTR sequence; **S1.** male CTTTA haplotype 3’UTR sequence; **S2.** male CTCTA haplotype 3’UTR sequence; **S3.** male CCTTA haplotype 3’UTR sequence; **S4.** male TTTTA haplotype 3’UTR sequence; **S5.** female CTTAACA haplotype 3’UTR sequence; **S6.** female TTCAACA haplotype 3’UTR sequence; **S7.** female TTTAACA 3’UTR sequence; **S8.** female 6568_6571del 3’UTR sequence; **S9.** female 7338_7344del 3’UTR sequence; **S10.** female 7065_7066insG 3’UTR sequence; **S11.** 50 microRNAs expressed in human hippocampus; **S12.** 50 microRNAs expressed in human frontal cortex; **S13.** 50 microRNAs expressed in human cerebellum; **S14.** 50 microRNAs expressed in human midbrain. (ZIP 403 kb) Additional file 3: Supplemental Figure 1. The binding types (8mer, 7mer-A1, et al) of the microRNA-mRNA (3’UTR) conserved binding sites from four data pools of CNS. a, shows the conserved sites and site types in the hippocampus data pool. Five types (offset-6, 6mer, 7mer-m8, 7mer-A1, 8mer) of conserved sites within the dark–light column and 11 genotypes 3’UTR was illustrated in the histogram. b, shows the conserved sites and site types in the frontal cortex data pool. c, shows the conserved sites and site types in the cerebellum data pool. d, shows the conserved sites and site types in the midbrain pool. (TIFF 2.43 mb)
