# Supplementary material for: The genetic variants in 3’ untranslated region of voltage-gated sodium channel alpha 1 subunit gene affect the mRNA-microRNA interactions and associate with epilepsy
Source: BMC Genet. 2016 Jul 29;17:111. doi: 10.1186/s12863-016-0417-y (PMC4966731; doi:10.1186/s12863-016-0417-y)
Supplement: Additional file 1: — Supplemental material. (DOCX 116 kb) [file 12863_2016_417_MOESM1_ESM.docx]

**Supplemental material M1**

**Methods**

**Genotyping**

The whole blood of study subjects was obtained from peripheral arteria brachialis with EDTA anticoagulation. A 2ml blood sample was then added with 6ml red blood cell lysis buffer (NH_4_Cl 8.3g, KHCO_3_ 1 g, EDTA 37mg in 1L H_2_O), gently mixed, centrifuged at 2000xg for 5 minutes. The white blood cell pellet was washed 2 times with phosphate buffering saline, diluted in 2ml white blood cell lysis buffer (0.1% SDS, 7.5M guanidine hydrochloride) and 100μg/ml protease K (Merck, Germany), heated to 60°C for 30 minutes. After the solution was cooled down and centrifuged at 12000xg for 10 minutes, the supernatant was moved into new tubes for DNA precipitation, mixed with 4ml cold pure ethanol and 600µl 3M acetate sodium, centrifuged at 10000xg for 10 minutes. The tiny white pellet at the bottom of the tube was washed with 75% ethanol twice (centrifugation at 7500xg for 5 minutes), moderately dried, and solved with TE buffer (10mM Tris, 1mM EDTA). The final concentration of the DNA solution ranged from 20 ng/μl to 200 ng/μl (measured by NanoDrop 1000) and was directly applied in PCR experiment for target fragments replication (3’UTR in *SCN1A* gene). The four pairs of primers for genomic DNA replication were listed below, wp615-5’-TGATC TGACC ATGTC CACTG C, wp616-5’-CCCTC ATGCA AACCA CGAC (680bp); wp617-5’-TTTTG TAAAC GAAGT TTCTG TTGAG, wp618-5’-GAAAC CAGAT ACAGC AGCAT GG (732bp); wp619-5’-TGTAG AGTGC AAGCT TTACA CAGG, wp620-5’-GAATC GTGAA CCTAT TTTGC TCC (601bp); wp621-5’-CACAA TCACT TTTCT TACTT TCTGT CC, wp622-5’-CCTTC TCCCC CAATT TGTAA TG (660bp). The PCR (TransStart Fast*Pfu*, High Pure dNTPs, TransGen Biotech Co, Beijing, China) products were sent to BGI Guangzhou Office for sequencing (ABI 3730xl sequencer). If the sequencing files indicated the deletion or insertion mutations (Chromas Lite 2.0), we purified the PCR fragment with QIAquick Gel Extraction Kit (QIAGEN 28704, MD, USA), linked (T4 DNA Ligase, TransGen Biotech Co) the fragments into pEASY-Blunt Cloning Vector (CB101-01, TransGen Biotech Co), transfected the linked vectors into Trans1-T1 Phage Resistant Chemically Competent Cell (CD501-02, TransGen Biotech Co), recovered the T1 cells with SOC media under 37°C and 200 vortex/min in 1 hour, swiped 100ul T1 cell precipitation on the LB Broth agar, after 8-12 hours under 37°C, selected the IPTG/X-gal white clones to dilute with 10μl LB Broth media, used 1-2μl of the clone-diluted medium as the plasmid DNA template for PCR evaluation (M13 forward primer-wp616/wp618/wp620/wp622, EasyTaq DNA Polymerase, TransGen Biotech Co. Beijing, China). The positive PCR products with correct fragment length were sent to BGI Guangzhou Office for sequencing data. After the PCR evaluation, the rest of 8-9μl clone-diluted medium of correct clones was added into 3 ml LB broth and cultured under 37°C, 200 vortexes/min, in 10-16 hours for plasmid replication.
