# Supplementary figures and images for: The genetic variants in 3’ untranslated region of voltage-gated sodium channel alpha 1 subunit gene affect the mRNA-microRNA interactions and associate with epilepsy
Source: BMC Genet. 2016 Jul 29;17:111. doi: 10.1186/s12863-016-0417-y (PMC4966731; doi:10.1186/s12863-016-0417-y)

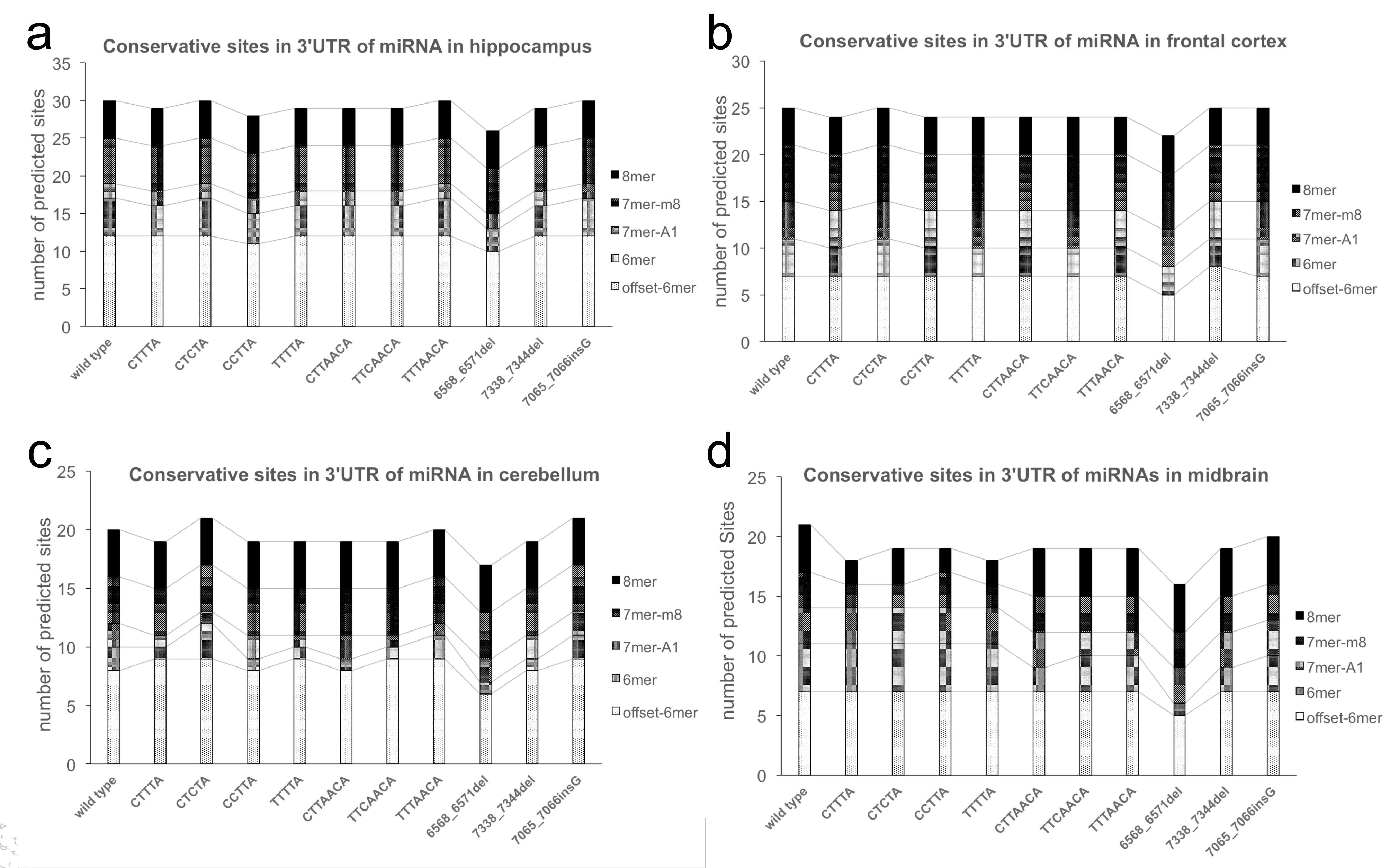

Supplement: Additional file 3: — Supplemental Figure 1. The binding types (8mer, 7mer-A1, et al) of the microRNA-mRNA (3’UTR) conserved binding sites from four data pools of CNS. a, shows the conserved sites and site types in the hippocampus data pool. Five types (offset-6, 6mer, 7mer-m8, 7mer-A1, 8mer) of conserved sites within the dark–light column and 11 genotypes 3’UTR was illustrated in the histogram. b, shows the conserved sites and site types in the frontal cortex data pool. c, shows the conserved sites and site types in the cerebellum data pool. d, shows the conserved sites and site types in the midbrain pool. (TIFF 2.43 mb) [file 12863_2016_417_MOESM3_ESM.tiff]
